# Spectral Analysis and Information Entropy Approaches to Data of VLF Disturbances in the Waveguide Earth-Ionosphere

**DOI:** 10.3390/s22218191

**Published:** 2022-10-26

**Authors:** Yuriy Rapoport, Volodymyr Reshetnyk, Asen Grytsai, Volodymyr Grimalsky, Oleksandr Liashchuk, Alla Fedorenko, Masashi Hayakawa, Andrzej Krankowski, Leszek Błaszkiewicz, Paweł Flisek

**Affiliations:** 1 Space Radio-Diagnostic Research Centre, University of Warmia and Mazury, 10-720 Olsztyn, Poland; 2 Physics Faculty, Taras Shevchenko National University of Kyiv, Kyiv 01033, Ukraine; 3 Main Center of Special Monitoring, National Space Facility Control and Tests Center, State Space Agency of Ukraine, 01010 Kyiv, Ukraine; 4 Centro de Investigación en Ingeniería y Ciencias Aplicadas, Universidad Autónoma del Estado de Morelos, Cuernavaca 62209, Mexico; 5 Space Research Institute National Academy of Sciences of Ukraine and State Space Agency of Ukraine, 01010 Kyiv, Ukraine; 6 Hayakawa Institute of Seismo Electromagnetics Co., Ltd. (Hi-SEM), Tokyo 182-0026, Japan

**Keywords:** VLF signal, Japanese VLF network, waveguide earth–ionosphere, Fourier analysis, Shannon entropy, terminator passage, modulation, acoustic-gravity waves, Brunt–Väisälä period

## Abstract

Very low frequency (VLF) signals are considered as an important tool to study ionosphere disturbances. We have studied variations in signal amplitude of the Japanese JJI transmitter received by a network of eight Japan stations. The distinctions between characteristics of daytime and nighttime disturbances are considered. Signal processing based on spectral analysis is used to evaluate typical periodicities in the VLF signals in the time range from minutes to hours. In particular, we have retrieved quasi-wave oscillations of the received signal with periods of 4–10 and 20–25 min, which can be associated with atmospheric gravity waves excited by the solar terminator, earthquakes or other reasons. In addition, oscillations at periods of 3–4 h are observed, probably, caused by long-period gravity waves. We also calculate the information entropy to identify main details in daily VLF variations and influence of solar flares. It is shown that the information entropy increases near sunrise and sunset with seasonal variation, and that solar flares also lead to the growth in information entropy. A theoretical interpretation is given to the typical features of ultra-low frequency modulation of VLF electronagnetic wave spectra in Waveguide Earth-Ionosphere, found by processing the experimental data.

## 1. Introduction

Propagation of radio waves near the Earth is determined by layers of ionized particles available at the heights from 50 km and upwards [1]. The layers traditionally named as D, E, and F form the ionosphere with maximal electron density at the heights near 250 km. Respectively, the waves reflect at the heights where the plasma frequency are equal to the own frequency of the waves. Waves with frequencies exceeding the maximal (critical) plasma frequency can travel towards the free space. In the opposite case, waves propagate in the waveguide of Earth–ionosphere (WGEI) with the possibility of several reflections. As a result, the corresponding signals can be received at large distances from a transmitter. Characteristics of the reflected waves are dependent on properties of the ionosphere that allow us to analyze indirect ionosphere disturbances of arbitrary origin [2,3,4,5].

### 1.1. Factors Affecting the Propagation of VLF Signals

During the propagation and reflection at very low frequencies (VLF), radio waves undergo scattering and absorption, which, of course, reduce their intensity depending on the distance to the source and the number of reflections. The Earth’s surface (continents, sea and ocean surfaces), and especially the ionosphere, are not ideal mirrors for VLF waves, some of which can penetrate upwards or transform into other types of ionospheric perturbations [6]. Therefore, VLF signals can serve as a kind of powerful probe that allows us to determine the state of the reflective surface. This property allows for track changes in the atmosphere and lower ionosphere, as well as in the underlying surface [7]. VLF waves are reflected at the heights of the ionospheric D layer creating a good possibility to study the state of plasma in that layer. The D layer is quite difficult to directly probe because it has a relatively low electron concentration, a specific ionic composition, and is located at the heights unreachable for altitudes by planes and balloons, while spacecrafts can directly explore much higher ionosphere layers. The main factors disturbing the ionospheric D layer are processes related to solar-terrestrial connections, such as solar flares, powerful magnetic storms, intense precipitation of auroral electrons from the Earth’s magnetosphere, and so on. The state of the lower layers of the ionosphere can also be affected by processes occurring at lower altitudes, namely in the troposphere and stratosphere and even on the Earth’s surface. Examples of such phenomena are powerful atmospheric gravity waves (AGWs), the passage of weather fronts, cyclones and hurricanes, possibly earthquakes, and so on. To some extent, the ionosphere can be seen as a mirror that reflects the processes above (primarily due to the influence of the Sun) and processes below, and the VLF waves are an important tool to study changes in the WGEI. The main problem is the correct separation of the effects of various phenomena on the propagation and reflection of VLF waves because, in reality, they all act simultaneously.

The main factor that forms the visible structure of the ionosphere is the solar radiation; ultraviolet and X-ray quanta are the main source of the ionization in the lower ionosphere [1]. Therefore, the distribution of electron concentration in the D layer has a clear daily variation, and, as a consequence, the reflection conditions (the height of reflection, the reflection coefficient, etc.) depend on the level of insolation. Even simple models can give the height of the VLF wave reflection depending on the solar zenith angle for the low-latitude ionosphere [8], where there are no significant perturbations of the electron concentration profile by other factors [9]. It is obvious that each specific trajectory of VLF signal propagation from transmitter to receiver will have its own parameters of scattering, so the daily course of the signal level may have some characteristic appearance for different paths.

The daily course of the VLF signal level on a particular path will vary depending on the season, as the level of insolation changes, as well as conditions in the Earth’s ionosphere and magnetosphere. Perturbations caused by various factors of terrestrial and space origin influence daily variations of the VLF amplitude. Some of the sources of disturbances of the received signal level lead to quasi-wave variations, while others have a clear one-time nature (increase or decrease of the signal level). One of the most energetic sources of near space disturbance is solar flares, which drastically increase the flux in the X-ray range, which in turn modifies the lower layers of the daily ionosphere around the planet in a relatively short period of time (from minutes to tens of minutes). During solar flares in the Earth’s ionosphere, sudden ionospheric disturbances (SIDs) appear [8]. In this case, the reflection coefficient of VLF waves from the D layer can change qualitatively (usually increases), which is well visible in the level of the received signal [10,11]. Thus, the VLF signal level can serve to monitor SIDs occurring on the path.

In addition to energy sources from above, which disturb the state of the lower ionosphere, there are other energy fluxes from below. A remarkable example of such disturbances is lightning striking the ionosphere. Powerful lightning can cause additional electron precipitation onto the lower layers of the ionosphere, which can be registered due to changes in the level of the reflected VLF signal on the propagation path [5]. In addition, the lightning itself is the generator of its own VLF waves.

The potential of using VLF data for earthquake prediction is discussed even recently by [12]. The authors considered the two-week period before the Kumamoto earthquakes in 2016, analyzing changes both in time and space. It is claimed that it is possible to determine the area of about 300 km, where the epicenter of the earthquake was located. The study of variations in VLF signals has been conducted by various methods in recent years [4,13,14]. In particular, a recent paper [15] analyzed possible earthquake predictors in Nepal in April 2015 using data series from various sources, including VLF signals and the total electron content (TEC). Note that, as proposed in [2,13,16,17], the perturbations of the characteristics of VLF waves in the WGEI can be considered as the ionospheric precursors of the most powerful earthquakes and, probably, the formation of hurricanes, respectively. Concerning the earthquakes, the corresponding mechanism of the seismo-ionospheric coupling has been proposed in [16,18,19,20,21]. The optimized method of searching for VLF seismogenic perturbations in the WGEI using the peculiarities of the solar terminator has been used in [22]. A method for reproducing the properties of AGWs in the mesosphere by fluctuations in the amplitudes of VLF radio signals on various European paths was developed in [23].

In [24], an approach was developed aiming to approximate the fluctuations of the neutral atmosphere caused by AGW propagation by measuring the amplitudes of radio signals on relatively short paths (with lengths less than 2000 km that correspond to the Japanese paths studied in this paper). The physical mechanisms of the influence of AGWs on the amplitudes of VLF signals were analyzed. It is shown that the AGW propagation affects the amplitude of radio waves mainly due to changes in the height of reflection. In the approximation of geometrical optics, relations were obtained that allow us to estimate fluctuations in neutral density and vertical displacement of the neutral gas volume at reflection heights from fluctuations in the amplitudes of radio waves. An influence on the atmosphere and ionosphere by nonlinear AGWs caused by tsunami has been modeled theoretically in [25]; corresponding influence caused by ground-based acoustic generator has been established experimentally in [26] and theoretically in [27,28,29,30,31]. As shown in the review by [32], a study of VLF waves in the WGEI allows us to cover the wide range of the physical processes in the system of Atmosphere–Ionosphere, in particular “lightning-induced short term perturbations; extra-terrestrial radiation bursts; energetic particle precipitation events; solar eclipses; lower atmospheric waves penetrating into the D-region; sudden stratospheric warming events; the annual oscillation; the solar cycle; and, finally, the potential use of VLF narrow-band measurements as an anthropogenic climate change monitoring technique”.

We note an important methodological and practical consequence of the fact that wave excitations of the VLF and Ultra-Low Frequency (ULF) range in the ionosphere are considered as indicators of the impact of the most powerful sources of the most diverse physical nature, namely those determined by catastrophic phenomena associated with space weather, meteorological and ionospheric processes, and located above, below and inside the ionosphere. Despite all their physical diversity, it is natural to point out the most general approach to the phenomena, which is undoubtedly the synergetic approach [33]. Currently, a whole class of universal synergetic approaches is being developed, based, in particular cases, on analysis, which is of great importance for the determination of entropy and information [34,35,36]. The corresponding features of Shannon entropy [33] correspond qualitatively to the features of entropy for an open system in the case of self-organizing development of catastrophic processes, in particular. This is shown, for example, for the earthquake in L’Aquila, Italy [34]. In our work, we develop a technique for applying the Shannon entropy to study the dynamics of VLF excitations in the WGEI in connection with the impact of the solar terminator and solar flares on the ionosphere.

Radio waves at frequencies lower than the maximum plasma frequency of the ionosphere F2 layer ($\nu_{hmF2}$) cannot pass freely through the ionosphere. Such radio waves, being generated near the surface or in the Earth’s atmosphere, are not able to go beyond the upper ionized layers of the atmosphere and propagate in the WGEI. This feature of radio waves at frequencies lower than $\nu_{hmF2}$ allows them to be used in man-made activities.

Nowadays, there are hundreds of radio transmitter stations of very low frequency and low frequency (LF) bands in the world. Their main task is to transmit navigation tags and signals of the exact time, and sometimes they are used for communication (especially with submarines because VLF radio waves can penetrate into the seawater to depths of tens of meters). Many receiving antennas have also been built in the world, which record the radio signals of well-known transmitting stations. VLF signals can serve as an indicator of the state of the ionosphere, atmosphere, and surface of the planet.

### 1.2. Main Goals of the Work

In this work, we study features which are typical for VLF data received by the Japan network of receivers. Both spectral characteristics and influence of separate events are analyzed mainly from measurements of the VLF signal amplitude. Our idea is to determine the most significant periods of disturbances in VLF characteristics with identification of their possible causes. We will use the great advantage of possessing data from the set of VLF stations (in fact, set of VLF sensors) and will compare their responses, such as an influence of hydrodynamic oscillations (caused by AGW) on the processes of VLF wave propagation in the WGEI with the corresponding modulation of the VLF spectra with the periods characteristic for AGW waves. In addition, the reaction of the receiving signals on usual natural phenomena as terminator passage and solar flares is also considered.

When processing the experimental data, the features of the modulated ULF VLF spectra are found. For their interpretation: (a) the very presence of ULF modulation of the VLF EMW (electromagnetic wave) spectrum propagating in WGEI is qualitatively explained; (b) ULF modulation spectra of VLF EMW in WGEI, which we singled out in this work on the basis of experimental data, are compared with the results obtained from models of resonant global atmospheric oscillations.

The structure of the work is as follows: After the Introduction, in Section 2, we describe methods used in the paper emphasizing the Fourier analysis and Shannon entropy. Section 3 includes an analysis of daily variations in the VLF signal amplitude with identifying variations at the terminator passage and solar flares. Spectral characteristics are also studied in Section 3. An interpretation is given of the spectral features of the modulated ULF spectra of VLF EMW in WGEI, found by processing the experimental data. Details of this interpretation are included in our article in Appendix A, appropriate references to which are included, in particular, in Section 3. Section 4 is devoted to approaches in using information entropy to VLF amplitudes, and Section 5 considers reaction of information entropy on terminator passage and its daily changes. Section 6 includes the discussion and finally Section 7 presents conclusions obtained in the present work.

In the future, the approaches and methods realized in the study can be useful to solve different problems of VLF reaction on disturbances “from below” and “from above”. In particular, disturbances of the lithospheric and atmospheric origin connected with earthquakes, hurricanes, and thunderstorm activity can be also analyzed. More specific study of solar and geomagnetic influence with attention to statistical properties of VLF changes is also needed.

## 2. Data and Methods of Analysis

### 2.1. VLF Data

**Figure 1:**
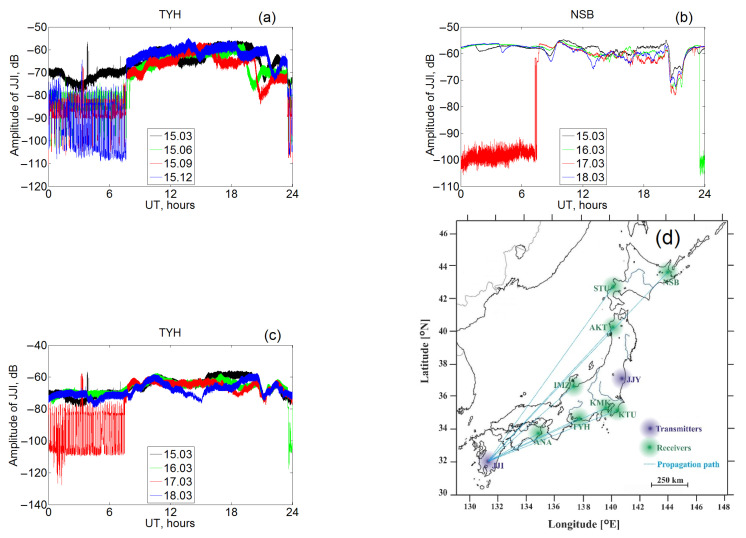
Diurnal variations of the JJI transmitter VLF signal amplitude: observations at TYH station on 15 March, 15 June, 15 September, and 15 December 2015 (**a**); NSB and TYH stations, 15–18 March 2015 (**b**,**c**). Amplitude values are in decibels. The location of the transmitter and eight receivers is shown in (**d**).

This paper includes the results of the analysis of VLF signals that propagated over Japan. We have used data from the Japan VLF/LF network [16,37]. This network was in operation for the last years. The network includes eight VLF receiving stations, namely (see their geographical coordinates in Table 1 and location in Figure 1, lower panel, right): Akita (abbreviated AKT), Anan (ANA), Imizu (IMZ), Kamakura (KMK), Katsuura (KTU), Nakashibetsu (NSB), Suttsu (STU), and Toyohashi (TYH). The transmitter and all receivers are located in different parts of Japan. The shortest JJI–ANA path was about 400 km long, and the longest JJI–NSB path is 1800 km. It should be noted that most propagation paths pass near the coast, sometimes crossing the coastline several times. All of these stations have identical receivers registering simultaneously the narrowband modulated signals in the range of 10–40 kHz from several transmitters. The vertical electric field component is measured by an electric rod antenna. VLF/LF data from the Japanese network are divided into daily files for each receiving station. The file contains observational data, particularly time, signal amplitude. Time resolution of the receiving signals can be ranging from 50 ms to 60 s, but the data with a sampling frequency of 1 Hz are used. There are two Japanese VLF transmitters. One of them is located in Ebino, Kyushu (denoted as JJI in Figure 1, lower panel, right; its geographical coordinates are $\varphi =32.08^{{}^{\circ}}$ N, $\lambda =130.83^{{}^{\circ}}$ N, and the frequency is 22.2 kHz. The other Japanese transmitter is JJY (frequency of 40 kHz) located in Fukushima (geographical coordinates are $37.37^{{}^{\circ}}$ N, $140.85^{{}^{\circ}}$ E); it is also shown in Figure 1, lower panel, right. In this work, we have used only the data of the JJI transmitter during a period from January 2014 to February 2017, i.e., for a period of about three years.

We consider specific properties of VLF signals including their typical periods and variations under the influence of such factors as solar terminator passing and solar flares. The periodicity is studied using spectral methods, in particular, by the Fourier analysis. VLF changes at separate events are processed by the Shannon entropy. The last one is used as a suitable instrument of searching synergetic processes in the open dynamic system Atmosphere–Ionosphere–Magnetosphere [33,34,38,39,40,41,42,43].

We consider applications of some methods of processing signal characteristics on an example of available measurements of the JJI transmitter amplitude, which were carried out at eight stations with designations AKT, ANA, IMZ, KMK, KTU, NSB, STU, TYH (Table 1). Data from these stations have been repeatedly used in the study of the VLF signal propagation in the ionosphere, in particular, when considering the possible effects of earthquakes [44,45].

### 2.2. Methods of Analysis

For spectral analysis, in particular, of VLF signal amplitude, the discrete Fourier transform is used:

(1) $$g\left(j\right)=\sum_{k=0}^{n-1}f\left(k\right)e^{-\frac{2\pi jk}{n}i}\;$$

with the following calculation of the modulus g(j). The result obtained corresponds to the frequency $\nu_{j}=j*\nu_{min}=\frac{j}{T}$ (*T* being the length of the considered time interval). Attempts to detect oscillations in time ranges longer than a day are difficult via the largest harmonics in the spectrum caused by the terminator and the daily variations of the signal. Therefore, the procedure of detrending the signal was used. To realize this, the average daily signal was subtracted from the studied signal (the example is shown later below). We centered the interval of time on which the average daily variation was calculated on the analyzed day using windows from 5 to 15 days. Let *i* be the number of the day, and *j* be the number of the second one. Accordingly, the detrended value is:

(2) $${\tilde{a}}_{ij}=a_{ij}-\frac{\sum_{k=i-s}^{k=i+s}a_{kj}}{2s+1}\;$$

where 2*s* + 1 specifies the number of days on which averaging is performed. The use of such differences is typical in processing VLF data. For example, in the paper by [45], the monthly mean is subtracted from the amplitude. In [13], the monthly value calculated on the basis of quiet (in terms of geomagnetic conditions) days is applied for the subtraction. At the same time, it is logical to assume that centering on the studied day is more justified to avoid seasonal variations than using the average for a calendar month. In particular, this approach with a range of ±15 days was used in [44] with the calculation of moving averages. The difference between the real and average value of the amplitude is used to search for predictors of earthquakes [46,47].

The wavelet analysis is used to determine time variability of spectral components. A general scheme is described as [48]:

(3) $$F\left(\tau ,s\right)=\frac{1}{\sqrt{s}}\int_{-\infty }^{\infty }f\left(t\right)\psi \left(\frac{t-\tau }{s}\right)dt\;$$

where f(t) is the analyzed signal, ψ is mother wavelet, τ is the time shift and *s* is the scale parameter. In this work, the Morlet function was chosen as a mother wavelet:

(4) $$\psi \left(t\right)=e^{\frac{-t^{2}}{2}}*e^{iat}\;$$

This combination of Gaussian and sinusoid is a useful tool to separate out a quasi-harmonic oscillation localized in time. The scale parameter is connected with period by interrelation [48]

(5) $$T=\frac{4\pi *s}{a+\sqrt{2+a^{2}}}\;$$

In this work, the parameter *a* was taken as 6, which corresponds with T≈1.23∗s. To improve the detection of periodical oscillations, nighttime data were processed because of their more regular changes [38,44]. During the course of their wavelet transform, the elimination of a polynomial trend was utilized with calculations of the trend by the least-squares method [49].

The information entropy is relevant to describe the synergetic processes in opened dynamical systems such as Earth–Atmosphere–Ionosphere–Magnetosphere (Shannon entropy [39,40]). This approach is used to various processes, in particular, in the analysis of the seismic activity [41], where the aim is to determine the quantity of information carried by the signal. When this method is applied, the analyzed interval is divided into subintervals to calculate the probability of the studied characteristics to be in each of these subintervals. The probability is considered as a relative number of experimental values, which fall into a respective sub-interval. Then, the information entropy (information on one value) is as [33]:

(6) $$H=-\sum_{i=1}^{n}p_{i}\log p_{i}\;$$

The logarithm base is not of fundamental importance in the characterization of variations, affecting only the obtained absolute numerical results. From Equation (6), we conclude that the entropy will be zero if all values are placed in a single interval; instead, the maximum value is achieved for a uniform distribution with the same number of results in each subinterval. It follows from the above that, generally speaking, the result should depend on the division into intervals. At the same time, it is possible to offer different practical approaches. In particular, one possibility is in the partition performed with the use of the maximum and minimum values of the studied value for a necessary period of time (for example, day). Otherwise, the range is set based on some general considerations for the entire study period (e.g., a month). However, the representation for continuous data is used in the form:

(7) H=−∫p(x)logp(x)dx

Taking into account this relation, the choice of small intervals is justified, i.e., there must be a significant number of them. In the following, a preliminary analysis of the factors influencing the propagation of VLF and LF waves is performed. According to observations, typical periods of disturbances in the VLF amplitude, which appear during their propagation in the WGEI, have been identified.

We use a linear correlation coefficient to analyze similarity between two data series. The Pearson’s correlation coefficient is calculated as

(8) $$r=\frac{\sum_{i=1}^{n}\left(a_{i}-\bar{a}\right)\left(b_{i}-\bar{b}\right)}{\sqrt{\sum_{i=1}^{n}{\left(a_{i}-\bar{a}\right)}^{2}}\sqrt{\sum_{i=1}^{n}{\left(b_{i}-\bar{b}\right)}^{2}}}\;$$

where *a* and *b* are two data series, each of them consists of *n* elements.

## 3. Daily Variations of VLF Signal Accounting for Influence of Terminator

Note that amplitude jumps can occur or not occur at any station at any season of year. This is illustrated in Figure 1, which shows the daily curves of the signal amplitude at the TYH station from the JJI transmitter for four days with a three-month interval. In this case, only the day in March does not show sharp amplitude jumps; instead, the curve for 15 December has a long range with sharp and frequent amplitude variations by 25–30 decibels (dB). For 15 June and 15 September, amplitude decreases are registered less frequently, but they are present. It should be noted that, in the nightly period, a sharp decrease in the received signal was not observed, so the causes of the phenomenon, whatever they may be, are realized only during the day. Accordingly, in the analysis of night data, the effect of a sharp short-term amplitude decrease is not as harmful as in the analysis of the entire daily curve. All data are used to study daily variations, and Shannon entropy is quite suitable as an instrument, complementary to spectral analysis because it gives a possibility to consider data with sharp jumps.

It is necessary to mention that the local time at Japanese stations differs from the world time used in the measurements by approximately 9 h (see the longitudes in Table 1). Therefore, regularly observed changes near or shortly after 20 UT correspond to morning events close in time to the passage of the terminator. These variations in the morning amplitude on the daily curves have a characteristic shape starting from a noticeable decrease (∼15 dB) with the following increase, which is approximately twice less. The beginning of the changes undergoes expected seasonal variations registering about 19 UT in summer and 22 UT in winter.

We will also analyze the changes in the amplitude that were registered during 15–18 March 2015. It is worthwhile to note that the daily cycle for these days has different shape at the studied stations. There are two main types of anomalous variations, namely a decrease in amplitude at the end of the day on 16 March and staying at this level for several hours on 17 March; there are rapid changes of unknown origin in the specified period of time. There is no evidence that these anomalies are connected with The St Patrick’s Day geomagnetic storm observed later on 17 March. Examples of the variations on 15–18 March at two stations are presented in Figure 1. Figure 1 also contains the map showing the location of the stations presented in Table 1.

It is common in all cases (eight stations in total) that a sharp decline is observed until midnight on 17 March (UT), which corresponds to the morning in Japan. This decline is very strong compared to the usual daily variations, reaching 40–50 dB. Despite the recording of the signal at all the stations, there are doubts that it is of natural origin and is not related to the characteristics of the transmitter. Significant differences in amplitude at individual stations within minutes (Figure 1) are most likely due to the instability of the receiving equipment. All aforementioned features do not influence the use of these data to the following studies with spectral methods and Shannon entropy.

**Figure 2:**
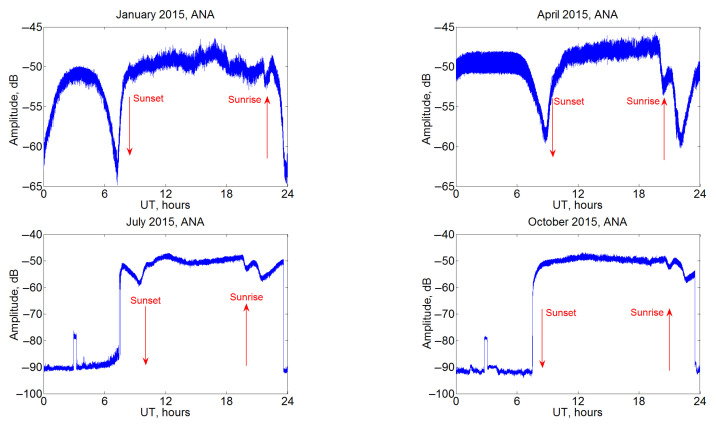
Monthly average variations of JJI VLF amplitudes at the ANA station in January, April, July and October 2015; median values for each second for all days of the month are used for the calculation. Local sunset and sunrise hours are indicated with red arrows.

**Figure 5:**
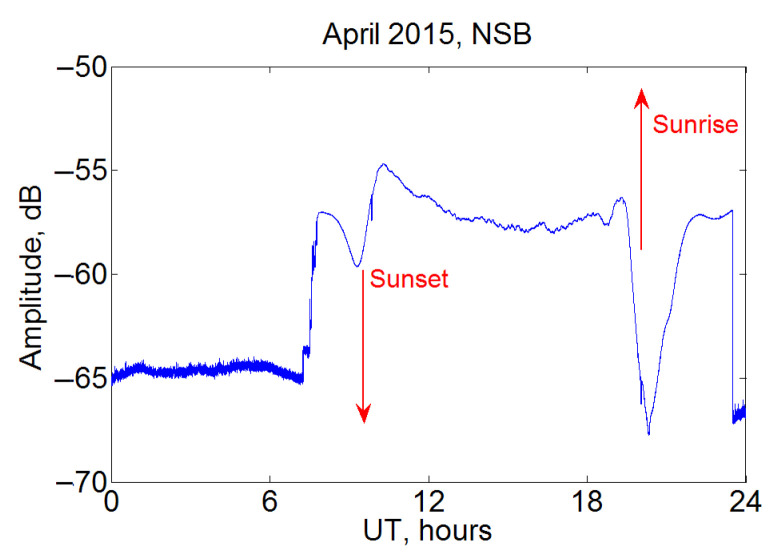
Example of average daily signal variation for NSB station, April 2015, JJI transmitter.

Seasonal changes in the daily variations of the VLF signal amplitude can be seen from Figure 2, which shows the monthly average values for the ANA station in different seasons of 2015. The amplitude range is from 15 dB in January to 40 dB in October; mostly, there is a clear difference between a higher signal level at night (about –50 dB in all cases) and a lower one at day. A slightly more complex pattern is observed in January, when the minimum amplitudes are reached in the morning and evening, and during the day the signal first increases with a maximum around noon (which corresponds to about 3 UT), and then decreases. The evening changes in amplitude in these examples are different, but the morning ones are always quite complex, with at least one local minimum, which precedes the daily decrease. The signal rise around the local noon (near 3 UT), which is clearly visible on three of the four plots (April, July, October), looks like an artifact. Nonetheless, it is not significant for the daily cycle. Two approaches to average values, arithmetic mean and median, were used to cross-check the results. Predominantly, daily variations obtained by the two methods are very similar. Note also that, when using the median values instead of the arithmetic mean, the April curve becomes generally similar to January, and the moments of sunrise and sunset are separated more clearly. At the same time, however, the scatter of values increases, especially for daily time in July and October, when it becomes difficult to analyze the corresponding dependencies using the median values. In the following (Figure 2 and Figure 5 below), we use only median values because arithmetic means show non-physical jumps of amplitude. Note that the general regularities of the curves are maintained for all stations.

**Figure 3:**
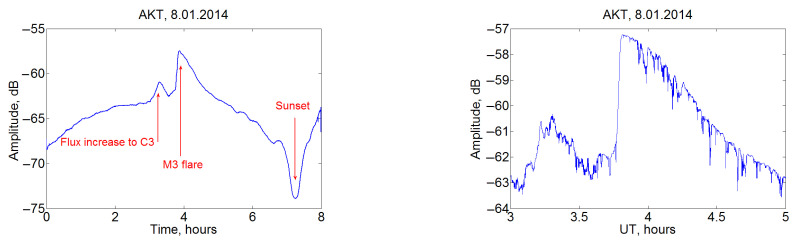
Example of SID events in the signals received by the AKT station during the solar flare on 8 January 2014: VLF amplitude in 0–8 UT with 10-min moving average (**left**) and the signal without processing in 3–5 UT (**right**). Moments of C3 and M3 solar flares are shown by the upward red arrows, and sunset is indicated by the downward red arrow.

In the following, we will focus on changes in the signal amplitude. The diurnal variations of the VLF signal level have a characteristic appearance, although the shape is specific to each path, and there is a significant seasonal change. Various disturbances are imposed on the diurnal trend, which are caused by processes of solar origin as well as the phenomena in the magnetosphere/ionosphere and in the lower atmosphere or on the underlying surface. An example of powerful events that affect the entire Earth’s atmosphere can be solar flares. One of the most noticeable disturbances are SIDs, which significantly affect the VLF wave propagation when the propagation path is in the illuminated hemisphere. An example of the registered changes in the VLF signal level at the AKT receiving station on 8 January 2014 is shown in Figure 3. Immediately before the M3 flare (03:39–03:54 UT) there was a significant increase in the X-ray solar radiation flux (to the level of C3, 03:04–03:20 UT ), which increased the level of ionization of the lower layers and led to an increase in the amplitude of the received signal (shown by the left arrow). The very beginning of the flare is marked by the middle arrow. After a solar flare, the level of the received VLF signal returned to undisturbed levels during an hour. The right arrow shows the time of sunset over the path.

**Figure 4:**
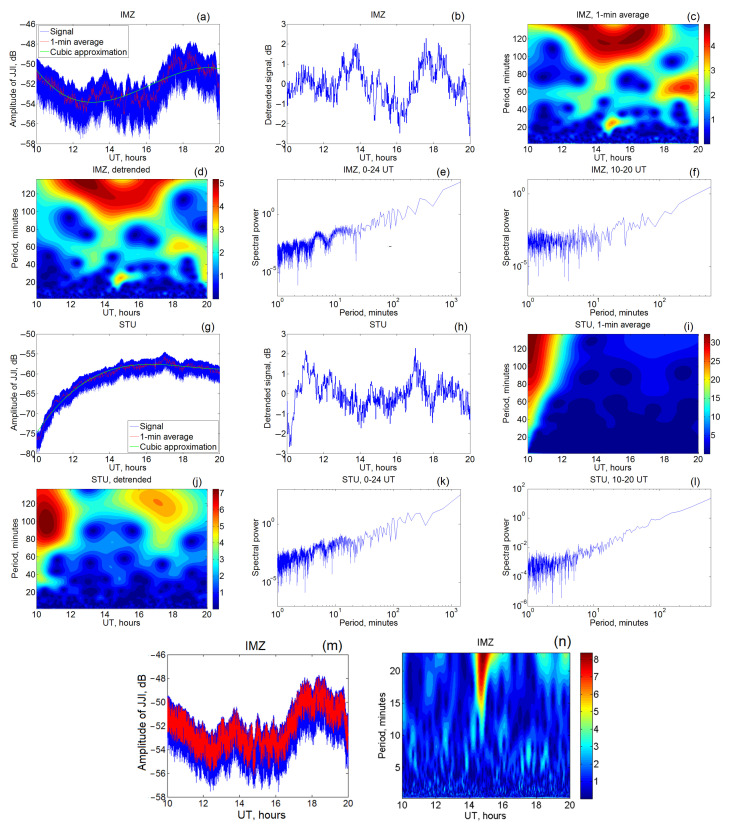
(**a**,**g**) Amplitude of the VLF signal (blue line), its 1-min averaging (red) and trend presented by cubic polynomials (green); (**b**,**h**) detrended signal; (**c**,**i**) wavelet transform after 1-min averaging; (**d**,**j**) wavelet transform after the detrending; (**e**,**f**,**k**,**l**) Fourier spectrum of the VLF signal, the whole day (**e**,**k**) and nighttime period (**f**,**l**) are presented. In all the cases, JJI transmitter signals received at IMZ and STU stations on 17 March 2015 are analyzed; (**m**) measured (blue line) and averaged on 10-s ranges (red line) JJI signal received at IMZ, 17 March 2015; (**n**) wavelet transform of the signal for minute periods.

Digital spectral analysis was performed for the received signal level at different stations of the Japanese network on different days. It was found that, in different seasons and even different days along a given path, the oscillations of different frequencies are excited, and there is not any permanent harmonic. The most significant permanent generator of wave activity is the terminator, and the wave activity is amplified at the time of its passage, which leads to the appearance of quasi-wave perturbations in the level of the received VLF signal. Variations of VLF amplitudes of registered signals at receiving stations IMZ and STU on 17 March 2015 and their spectra are presented in Figure 4a–n. On 17–18 March 2015, a powerful magnetic storm occurred, when the DST index reached –222 nT (Kp-index was about 8).

Nighttime VLF variations are typically without any sharp jumps which are specific for daytime measurements (see, for example, Figure 1, left part). Therefore, nighttime data were chosen to analyze periodicity in the VLF disturbances (Figure 4). We select a time range from 10 UT till 20 UT, which is between 19 UT and 5 UT in Japan. Typical VLF amplitude variation contains a systematical change (trend), oscillations with different frequencies and noise reaching sometimes several decibels (Figure 4a,g, the example for 17 March 2015; the variations can be disturbed to some degree by a geomagnetic storm). The level of the noise was decreased due to averaging over the consecutive 1-min time ranges. After this operation, the long-term trend was calculated by the approximation by the least-squares method. In the cases presented in Figure 4a,g, the signal was fitted by a polynomial of third degree. After subtraction of the polynomial, nighttime signal irregularly oscillates with changes of several decibels (Figure 4b,h). Figure 4m,n demonstrates separation of disturbances on time scales of several minutes. 10-second averaging is realized for this goal.

The wavelet transform by Equation (3) with the mother function of Equation (4) may be realized both before (Figure 4c,i) and after the described trend elimination (Figure 4d,j). In the case of the IMZ station (and of other stations despite of STU), a discrepancy between the two transforms is not significant. We can see several groups of periods consisting of their values 20–25 min, 60–70 min and nearly 120 min. It is worth emphasizing the local appearance of the respective disturbances that can complicate their search by common Fourier analysis. When the STU measurements are considered, large amplitude changes exceeding 20 dB were observed on 17 March 2015. Nonetheless, after the trend subtraction, oscillations are at the same level as for IMZ. Respectively, without the trend elimination, it is difficult to reveal any disturbances on the background of the wide maximum caused by a sharp amplitude increase at 10–12 UT. On the contrary, after the trend elimination, periods near 30 and 100–120 min are more visible on the plot.

In Figure 4, we also exhibit results of the Fourier transform with inclusion of both daytime and nighttime data (Figure 4e,k) and without daytime measurements (Figure 4f,l). Data for all periods have a more complicated spectral structure in comparison with daytime ones that partly can be explained by the role of sharp amplitude changes usually observed in daytime data (see, e.g., Figure 1). It is clearly seen from the Fourier transform in Figure 4 that the VLF signals are modulated by oscillations with periods of 10–20 min, which may be due to the presence of wave activity in the lower ionosphere. The intensity of these quasi-wave perturbations is distinct on the paths of different length, although in general the shape is close.

To reduce the impact of sharp jumps of unknown etiology, an algorithm for finding the average daily curve of signal level change was proposed. This approach is based on the analysis of measurements over a time range longer than a day (usually from several days to several months). For each second of the average curve, an average median value for the corresponding second of measurements from all days of the selected time period is calculated. For example, the first point of the curve is the average median value of the first seconds of all days selected for the analysis. Changes in the signal level during the day are characteristic of this type of signal [50] and are associated with the conditions of insolation of the lower ionosphere along the path.

In Figure 5, daily variations are shown for the NSB station located at Hokkaido; the JJI transmitter signal at Kyushu is used as earlier. Data from the Japanese station show a well-defined daily minimum in the signal with characteristic features at dawn and dusk. At the same time, the dawn effect is quite complex, with a two-moment decrease in amplitude with a return to the night level between these decreases. At sunset, the signal increases from −65 to −55 dB, also with a curve of complex shape.

**Figure 6:**
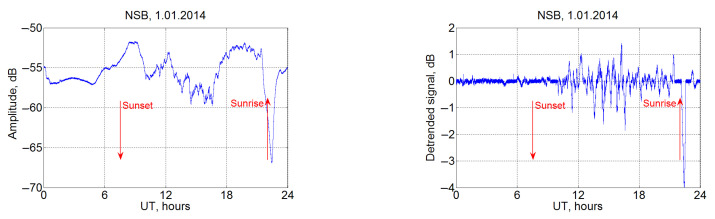
Amplitude variations (**left**) and detrended signal (**right**) by NSB station measurements, 1 January 2014.

An example of the detrended signals from the NSB station on 1 January 2014 is shown in Figure 6. Signal variations with a characteristic time of change of 0.5–2 h are quite noticeable, and the wave activity is most essential in the night ionosphere. However, a more detailed study demonstrates that these wave disturbances are available over the whole day, and solar radiation changes only the amplitude of these oscillations. In this case, the ratio of the amplitude of VLF signal variations at night and day is about one order of magnitude.

**Figure 7:**
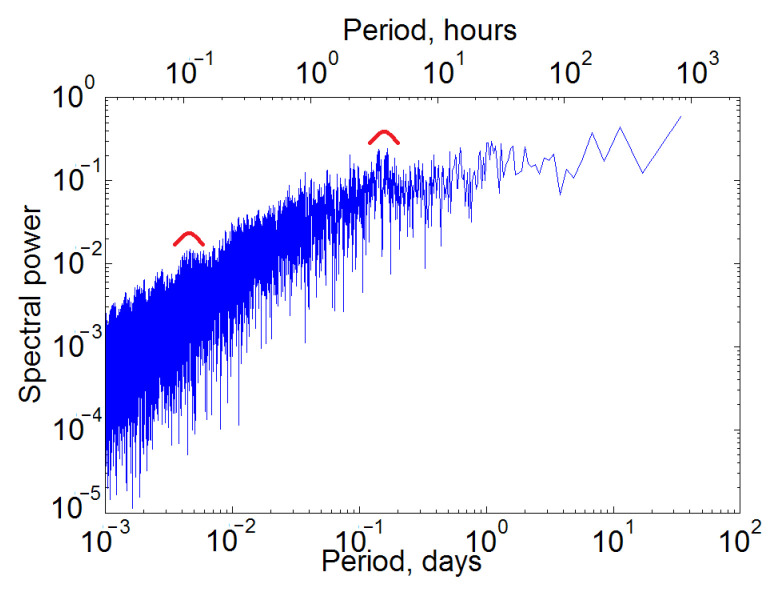
Fourier spectrum of variations in signal deviations from a daily mean at the NSB station for the period 22 January–24 February 2014. Maxima at the periods of 5–10 min and 3–4 h are shown by red lines.

The obtained detrended signals for each day can be joined into a longer time series with the application of spectral analysis techniques to it. An example of the spectra of variations in the VLF (detrended) signal is shown in Figure 7 for the NSB station during the period 22 January–24 February 2014. A noticeable increase in the spectrum is observed at the periods of 5–10 min (shown by the left red line in Figure 7), which may indicate the generation of oscillations with these periods. This period can be interpreted as connected with the Brunt–Väisälä frequency [51,52,53] near the Earth’s surface. The spectrum in Figure 7 demonstrates availability of fluctuations at the periods of about 20 min. These oscillations in the signal spectrum are not permanent, but they occur sometimes, so the power level of these perturbations is insignificant in the total spectrum for the month. For the JJI–NSB path, the presence of the wave activity with periods of 3–4 h was detected (shown by the right red line in Figure 7). These periods can be associated with the (long-period) gravity waves [51]. The vertical line indicates the period of one day, which still remains in the spectrum after the detrending operation because each day has some unique features, which is manifested in the signal spectrum. There are signs of a weekly period which can be caused by anthropogenical activity, but the studied interval is too short for clear conclusions.

Note that the results, mentioned above, on revealing the modulation of the VLF signals in WGEI with the frequencies typical for AGW in the atmosphere–ionosphere, correspond qualitatively to the fact that the AGW influence the reflection of VLF waves at the corresponding altitude from the ionospheric plasma [54].

We present the estimations illustrating the possible connection between the periods of oscillations characteristic for AGW and resonant properties of the atmosphere as the media of existence and propagation of AGWs. A detailed substantiation of this possibility of resonant modulation of VLF spectra by AGW frequencies is presented in Appendix A. For the convenience of readers, here we very briefly outline the essence of what is presented in Appendix A, namely: (1) interaction between AGW existing in the atmosphere and VLF EMW propagating in the WGEI can be connected with the generation in the ionosphere of the currents on the combinations $\omega_{VLF}\pm \omega_{AGW}$ of the frequencies $\omega_{VLF}$ of VLF EMWs and $\omega_{AGW}$ of AGWs. Such the current can be caused by two factors (see relations (A5), (A6) in Appendix A): (a) dragging of charged particles by means of AGWs against the background of ionospheric plasma with disturbances of charged particle concentration caused by VLF EMW; (b) motion of plasma particles with frequency of VLF EMW on the background of slowly varying plasma concentration, caused by AGW in the atmosphere–ionosphere; (2) AGW as global oscillations in the atmosphere–ionosphere are excited resonantly, and therefore relatively very efficiently, as the so-called reactive, or evanescent, modes [20,55,56,57,58,59]; an influence of the AGW packets containing such modes on the stable and unstable ionosphere has been investigated in [19,60] and [20,61], respectively. The estimation presented below would not cover comprehensively the all complex processes of the VLF modulation by AGW, outlined above. In the present paper, we restrict ourselves only with the demonstration of the fact that the obtained spectra of the modulation of VLF EMWs propagating in WGEI are quite compatible with the conditions of the resonant excitation of the global AGW modes. Then, we suppose that such resonant AGW mode excitations may be the reason of the most remarkable components of the revealed VLF spectra. Two main resonant reactive AGW modes [20,55,56,57,58,59] are the Lamb waves for which

(9) $$\lambda_{y}=c\tau ;\;k_{y}\equiv 2\pi /\lambda_{y};\;c^{2}=\gamma gH;\;H=k_{B}T/mg\;$$

and Brunt–Väisälä oscillations with frequency

(10) $$\omega \equiv \omega_{AGW}=\omega_{B};\;\omega_{B}^{2}=\left[\left(\gamma -1\right)/\gamma \right]\left(g/H\right)\;$$

In Equations (9) and (10), $\tau ,k_{y}$ and $\lambda_{y}$ are AGW period, wavenumber in horizontal direction and corresponding wavelength, respectively; c,g,H and γ are atmospheric sound speed, free-fall acceleration, atmospheric scale height and the adiabatic constant for the atmosphere; $k_{B},T$ and *m* are the Boltzmann constant, temperature of the (neutral) atmosphere and average mass of the atmospheric particles. Note that the temperature in the lower part of the atmosphere (zle100 km) does not change, in particular with altitude, remarkably compared to one in the thermosphere, respectively; the local approximation for the AGW field in the atmosphere can be used for the evaluations [62]. Respectively, it is supposed that the AGW velocity components $V_{y,z}$ are proportional to $\exp\left[i\left(\omega t-k_{y}y-K_{z}z\right)\right]$; here, $K_{z}=i/2H+k_{z}$, where the first term is connected with the presence of atmospheric stratification [62,63], $k_{z}=k_{z}^{\prime }+ik_{z}^{\prime \prime }$ is the effective vertical wavenumber of AGW with real and imaginary parts equal to $k_{z}^{\prime }$ and $k_{z}^{\prime \prime }$, respectively. For the propagating modes (in vertical direction), $k_{z}^{\prime \prime }=0$ [63], while for the resonant reactive modes (9), (10), $k_{z}^{\prime \prime }\neq 0$ [19,56,58,59,60]. In spite of the evanescent character of these modes, their impact on the ionosphere and VLF waves perturbations may be important in the case of the sources, distributed by the altitudes in the atmosphere–ionosphere in the wide amplitude range Δz;Δz is on the order of a few dozens of kilometres, namely Δz∼20 km for the strongest tropical cyclones [64,65] and for the sources forming before the strongest earthquakes [66]; seismogenic sources forming after the strongest earthquakes are powerful enough to provide the covering by the corresponding excited waves all the atmospheric altitude ranges up to the ionosphere [67]. Accounting for this, we will stress in our estimations on the most effectively excited global atmospheric Brunt–Väisälä oscillations and Lamb waves, in spite of their reactive character with the characteristic scale of evanescent decrease with the altitude of order $|k_{z}^{\prime \prime }{|}^{-1}∼H$. Consider these evaluations for the several characteristic spectrum components revealed during the data processing described above in the present Section 3.

The spectra maximum with the period $\tau ∼3\;h∼10^{4}$ s, accounting for that [63], c∼0.3 km/s may correspond, by the order of value, to the excitation of the Lamb wave with the half wavelength of order of 1500 km ($\lambda_{y}=c\tau /2$, compare with relation (9)), which corresponds to the characteristic dimension of the size of the horizontal projection of the terminator, see Figure 4n and [68]. Earthquake [18,69] or tropical cyclone [43] sources with the horizontal sizes (300–1000) km may excite the Lamb waves with the corresponding wavelengths (see Equation (9) and Appendix A) and the periods of order of (20–60) min (see Figure 4c–e,k and Figure 7). Then, the oscillations with periods τ∼6–7 min, presented in the VLF spectra (Figure 7), are probably excited global atmospheric Brunt–Väisälä oscillations (see Equation (10) and Appendix A, relations (A20)). Note also that the periods of the order of few minutes revealed in the VLF spectra (see Figure 4k,n and Figure 7) may characterise the AGW modes in the opened waveguide Earth–Thermosphere [70]. In accordance with the dispersion equation (based on the isothermal approximation [63] presented in Appendix A in the first line from the relations (A17), the dispersion of the AGW branch mode of waveguide Earth–Thermosphere is approximately:

(11) $$\omega /\omega_{a}∼\sqrt{1+[{\left(2H\right)}^{2}\cdot \left({k_{y}}^{2}+{k_{z}}^{2}\right)]};\omega_{a}\equiv c_{a}/2\cdot H;k_{z}∼\pi /L\;$$

In (11), L∼100 km is the effective width of the “Earth-Thermosphere” waveguide of the AGW. Using relation (11) and putting, for the rough estimations H∼ 8 km [63] and $k_{y}∼k_{z}$, yields τ=2π/ω∼ 4 min. By the order of value, such evaluations correspond to the similar theoretical results for AGW period presented in [71] and to the periods revealed in the spectra of VLF (see Figure 4n).

The more detailed analysis would be necessary to reveal the VLF modulation in WGEI by the strongly excited double resonant Brunt–Väisälä–Lamb oscillations [59], see relation (A21). This will be a subject of the next paper(s).

Note also that the oscillations with periods (1–2) min., which are the most pronounced among the AGW oscillations revealed in [54] from the spectra of VLF waves on the Germany–Serbia path, reflected from the upper boundary of the WGEI, are also presented in the spectra revealed from our data obtained as a result of the processing VLF data from the Japan paths (Figure 4n). In distinction to the data presented in [54], our data oscillations with periods (1–2) min are relatively weakly pronounced (Figure 4n).

## 4. Application of Information Entropy to Process VLF Signal Parameters

We will consider two approaches on Shannon entropy; the first of them requires dividing daily data into a respective number of intervals taking into account daily maximum and minimum amplitude. In the second approach, the values of maximum and minimum are determined from the monthly distribution and are the same for all the days.

In the example of March 2015 and the TYH station, we will analyze the results of using the Shannon entropy. The calculation is carried out in accordance with the formula of Equation (6). Firstly, we will consider the division into a fixed number of intervals determined by the maximum and minimum values for a particular day. Thus, for *n* intervals and daily limit values of amplitude $a_{min}$ and $a_{max}$, the intervals were confined by the numbers $a_{min}+k\Delta$, where *k* are the consecutive numbers from 0 to *n*; $\Delta =(a_{max}-a_{min})/n$. When using 3 to 80 intervals, it was found that visually the pattern changes slightly, and the features of variations at scales of several days are maintained. The minimum values were observed for 16, 26–27, and 30 March. The approaching of the Shannon entropy to zero was due to the values concentrated mainly in a small part of the intervals. With the described method of partitioning, this indicates the existence of abrupt changes of a short-term nature, in particular, outliers. In the case of VLF signals, rare amplitude variations with a characteristic time of several seconds are caused by noise effects, not natural processes. The pattern observed on 16 March (Figure 1) corresponds to this condition due to a large decrease in amplitude at the end of the day, i.e., there was a sharp asymmetry in the distribution. For 17 March, the asymmetry becomes more noticeable as the number of intervals increases because there are long periods of high and low amplitudes, and the intervals between these characteristic values contain almost no observational values.

For comparison, we describe the results of another approach, when the limits of the intervals are predetermined, not obtained from the actual maximal and minimal values. For March 2015, the values of −54 dB and −127 dB close to limit ones were taken, the same for all days. The plot of the amplitude for the whole month is not shown here, but the scale of variations can be imagined based on Figure 1 because the data for 15–18 March well characterize the variability in March in general. The maximum for 17 March significantly exceeded other values during the month, which is clearly different from the approach described in the previous paragraph, which was based on the local maximal and minimal values. The explanation is that, for a given size of the interval, the most homogeneous distribution will correspond to a large scatter of values when the scatter is not limited to individual points. This is close to the distribution realized on 17 March; see Figure 1. Instead, individual outliers have a weak impact, as is observed in the example of 16 March.

**Figure 8:**
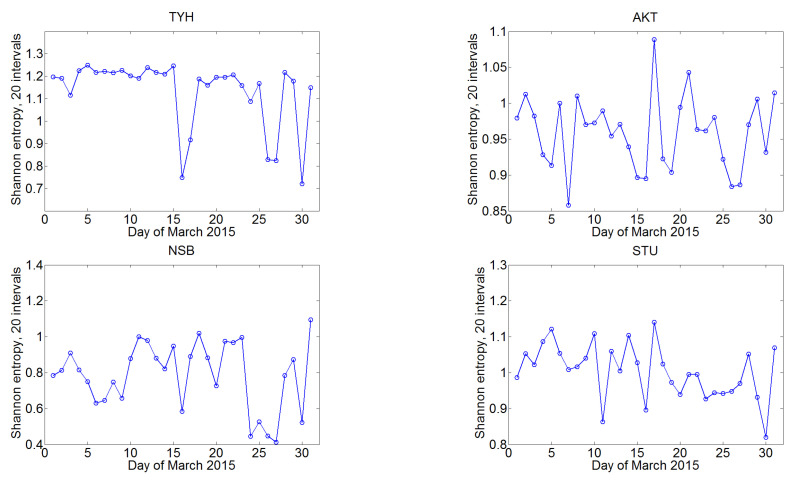
Shannon entropy in March 2015 for the amplitude of the VLF signal from the JJI transmitter to the stations TYH, AKT, NSB, STU.

It should be noted that there is a low level of agreement between the Shannon entropy variations for different stations within one month, and maximal and minimal values can be independent from one another. This is shown in Figure 8 on the example of measurements of the VLF signal amplitude in March 2015 at four of the eight stations of the Japanese network. For example, the decline on 16 March for the AKT station is absent at all, and the other three stations are different. In turn, at the AKT station, there is an increase in Shannon entropy on 17 March, absent in other cases. Many features are manifested only at one station; for example, the main maximum at the NSB is observed on 31 March. Pearson’s correlation coefficients calculated by the Formula (8) are also low. For example, 0.23 was obtained for STU and AKT.

## 5. Daily Variations of Information Entropy, Features of the Terminator Passage

To identify the possible relationship between the characteristics of the VLF wave propagation and different geophysical events, the information entropy of VLF signals during the selected events was analyzed. From such events, we have chosen the passage of the terminator over the path of VLF signals and solar flares that occurred during the day at the location of the propagation path.

To determine the influence of the terminator on the value of the information entropy in the VLF signals, the moments of sunrise and sunset over the Earth were found for the central part of the path. Obviously, the Sun cannot rise at the same moment over the entire path due to its latitudinal and longitudinal length and some uncertainty in the height. Radio waves propagate in the WGEI in a complicated way, so the time of the terminator passage is determined with some uncertainty, depending on the season, the actual location of transmitter and receiver, and other factors.

**Figure 9:**
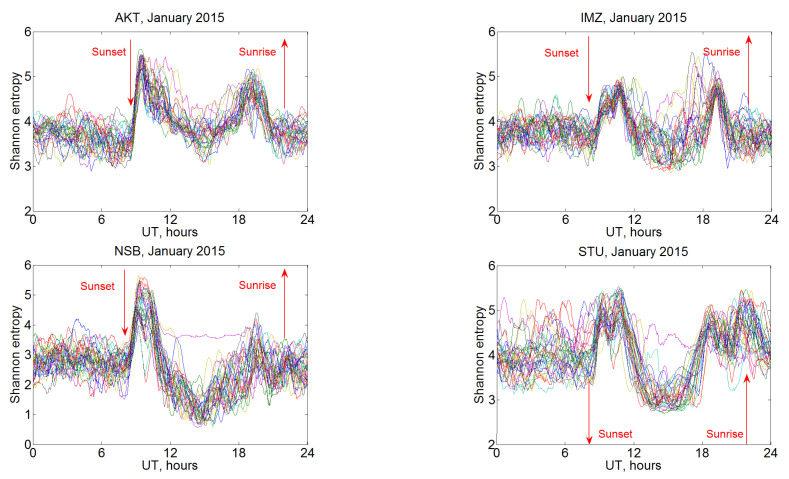
Example of daily variation of information entropy at the AKT, IMZ, NSB, and STU stations in January 2015; all days of the month are shown. Each curve shows an individual day.

The information entropy was determined from the VLF signal amplitude of individual stations. The time interval for which each value of information entropy in this section was calculated is 60 min, and the value was obtained by detrending with the elimination of moving average (2) with a time step of 1 min. The division into intervals of a constant length 0.1 was used. Examples of the information entropy daily variations at the four receiving stations are shown in Figure 9. All 31 curves for each day of January 2015 are given. It is seen that the information entropy at one time of day varies within certain small limits, but, for most curves, significant differences are not detected. However, there is a characteristic variation of the entropy curve during day detected at all the prescribed stations, with a first maximum near 10–11 UT (sunset) and the second one after 19 UT (sunrise).

**Figure 10:**
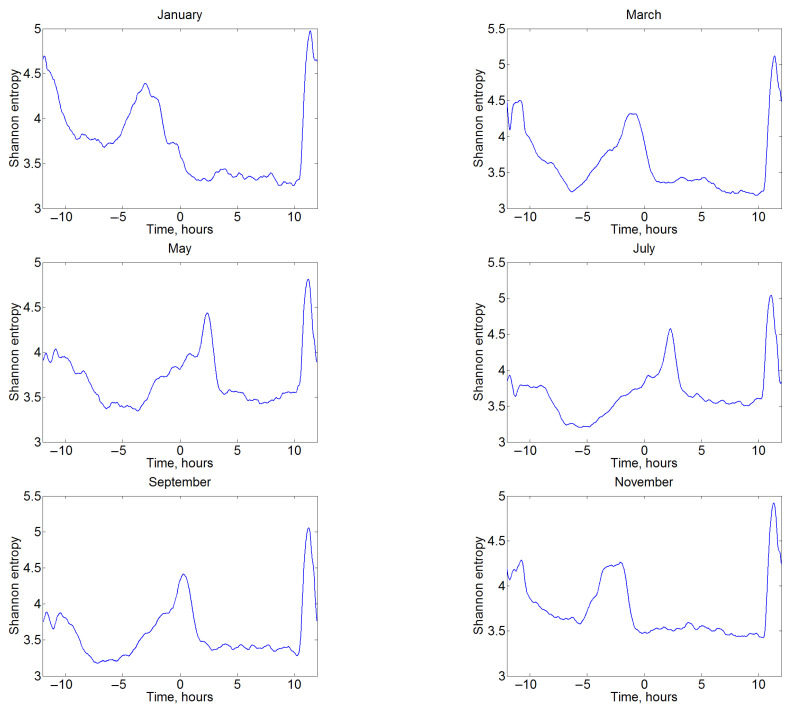
Variations in the monthly mean of information entropy at the AKT station in 2014, centered at the time of sunset. VLF amplitude data for January, March, May, July, September, and November are used.

It is obvious that the propagation of electromagnetic radiation in the WGEI can be influenced by many factors, all of which change the amplitude and phase of the signal, and hence, in the general case, the information entropy. In the analysis of the manifestation of the terminator passage in the information entropy of VLF signals, the method of superimposed epochs [72] was used, and where the epoch was identified as the moment of sunrise or sunset on the central part of the path. This approach partially eliminates other factors that are not related to the Earth’s diurnal rotation. Thus, all the presented curves of changes in information entropy (for example, as in Figure 10) were averaged with a shift at the time of sunrise or sunset. Consider, in particular, the main features of the average information entropy at the AKT station in January 2014 with centering at sunset over the path (time moment 0 on the abscissa). It is clearly seen that a significant increase in information entropy in January is observed before sunset (about by three hours earlier; the seasonal changes are considered below). A higher narrow peak at about 11 h (Figure 10) corresponds to a slightly expanded moment of sunrise (the expansion occurs due to centering at sunset, while the length of the day during the month obviously changed). However, it is interesting that information entropy begins to increase after sunrise and before sunset. This sensitivity to the passage of the terminator is revealed for all the observational stations, although there are some specific individual features in the magnitude and position of the peaks of the information entropy values.

The seasonal dependence of changes in information entropy during day is an interesting feature clearly visible in Figure 10 for different months at the AKT receiving station in 2014. It is noticeable that the passage of the terminator twice a day qualitatively changes the value of information entropy. At the same time, in the winter months, the information entropy has a peak before sunset (negative values on the abscissa axis) on the Earth’s surface below the path, while, in the summer months, it reaches an extreme after the terminator passage on the planet’s surface (positive values for hours). It can be assumed that this is due to the insolation of the upper atmosphere (layers D and E of the Earth’s ionosphere), where the effective reflection of electromagnetic waves from the ionosphere happens. The Sun in summer is not very deep below the horizon, and accordingly the duration of insolation at altitudes of 60–100 km will be much smaller than in winter.

## 6. Discussion

VLF signals can serve as an indicator of the state of the ionosphere, atmosphere, and surface of the planet. A large number of transmitting VLF stations permanently works on the planet that can be used to monitor near space. The analysis of VLF signals over Japan showed a significant effect of the level of solar insolation on the VLF and LF wave propagation. We have observed periods of 5–10 and 20–25 min, ∼1 h and 3–4 h, which can be associated with solar terminator passage, AGWs, i.e., disturbances in the ULF range, earthquakes and other atmospheric and lithospheric processes. In the spectrum of the detrended signal, the oscillations are detected:

- a noticeable increase is in the spectrum at periods of 5–10 and 20–25 min, which may indicate the generation of oscillations with these periods;
- at periods of 60–70 min and 3–4 h (long-period gravity waves);
- there are also indications of a weekly trend, which may be caused by the effect of anthropogenical activities on the atmosphere/ionosphere.

It is interesting that, as far as we know, this paper revealed, for the first time, an effect on the characteristics of the VLF propagation in the WGEI caused by AGW oscillations near the Brunt–Väisälä period (about 5–7 min), i.e., a period of fundamental atmospheric fluctuations. The influence of solar flares on the VLF wave propagation due to the SID formation near the path was confirmed.

The very effective method of spatio-temporal characteristics, in particular, of VLF perturbations in the WGEI appeared to be the combination of detrending and wavelet analysis with proper averaging. At four different receivers, oscillations of the same periods, in particular, 20 and 60 min, which are probably AGW perturbations [19,47], have been revealed at the nighttime with the use of the wavelet transform. This procedure was successful, in particular for the STU station only with detrending and after application of the wavelet analysis, while without detrending the corresponding oscillations have not been seen due to large systematical amplitude changes. These oscillations can be considered as “global”, by generalizing the fact that are observed at least in the frame of the wide region under consideration (Japan territory). A more detailed analysis of the reasons why these oscillations are observed at different stations at the different periods of time is highly required.

The diurnal cycle is most clearly visible in the variations of the VLF signal amplitudes. The available VLF data are characterized by sharp decreases in amplitude values, in correspondence of the varying frequency during the day, which complicates adequate processing. The testing of the information entropy was carried out, and its main features were clarified. Variations in information entropy at different stations in March 2015 are considered in detail.

Changes in the information entropy of VLF signals have been studied from measurements by the network of eight Japanese stations, and the nature of its response to terminator passage and solar flares has been established. Information entropy has been found to show maxima near sunrise and sunset, and the time of these peaks relative to the indicated moments changes with season. The effect of solar flares on information entropy has been previously established as the VLF amplitude increase (Figure 3), but this issue needs further studies.

The methods and technique for the monitoring of the state of the ionosphere, in particular, based on the analysis of the characteristics of VLF propagating in the WGEI would be also useful for the astrophysical research. In particular, this is concerned with astrophysical research based on radio telescopes such as the system of LOFAR (LOw Frequency ARray; [73]) working now in the MHz range as well as the actually constructed Square Kilometre Array (SKA) [74]. The point is that knowledge of the state of the ionosphere and the scattering of the corresponding radio waves on the ionospheric oscillations [75] should be taken into account for the precise analyses of the characteristics of radio waves registered by the radio telescope and excited by astrophysical objects. This would concern, for example, with the radio waves excited by pulsars, and the necessity to reveal the peculiarities of the radio wave spectrum connected to the astrophysical effects as such from those caused by the ionosphere. At the same time, radio telescopes, such as LOFAR, are useful for ionospheric research.

In the last several years, ionospheric monitoring became multi-instrumental, multiparametric, and synergetic [15]. This, the most general theoretical approach, is accompanied by a very general cybernetic approach, namely machine learning ([76]. The methods of signal processing used in the present paper (spectral methods and the method based on the application of the Shannon entropy) are quite compatible with machine learning. The unification of the data processing methods, described in the present paper, with the machine learning and accounting for the extra possibilities, connected with the presence of the set of VLF receivers (sensors), will be a subject of the next paper(s).

A qualitative explanation is proposed for the presence of ULF modulation in the VLF EMW spectrum propagating in the WGEI. It is shown that the perturbation at the combination frequency of VLF EMW and ULF AGW arises due to the charged particles dragging by ULF AGWs against the background of VLF perturbations of plasma concentration, as well as due to the motion of charged plasma particles in the VLF EMW field against the background of a slow change in the concentration of plasma, entrained by ULF AGW, propagating in WGEI. The amplitude of the disturbance at the combination VLF-ULF frequency is proportional to the product of the amplitudes of the VLF EMW incident on the region of the ionospheric disturbance and the ULF AGW. In this article, we limited ourselves to comparing the features of modulated ULF VLF spectra found by us, based on the processing of experimental data, with the results following from the model of resonant global atmospheric oscillations. As such global perturbations, evanescent (reactive) Lamb waves and global Brunt–Väisälä oscillations are singled out. The periods of such oscillations correspond to the spectral components that stand out in the VLF EMW spectra in WGEI as the results of ULF modulation. In particular, this concerns periods of the order of 6–7 min, 20–60 min, and 3 h. Modulating oscillations with periods of 4 min probably correspond to the waveguide type of global AGW disturbances, namely, their acoustic branch, excited in an open, but sufficiently efficient “Earth-Thermosphere” waveguide. In the following, the detail characteristics of external AGW sources and numerical modelling of the corresponding ionospheric response should be studied with a comprehensive analysis of combinatorial VLF-ULF disturbances in the ionosphere. It will also be interesting to investigate in future works the possibility of the influence of the doubly resonant evanescent (reactive) Brunt–Väisälä mode on the spectrum of ULF modulation of the VLF EMW [59]. In the future, the approach developed in this paper leads to a very important problem of constructing a combined synergetic EMW-AGW model of wave disturbances as communication agents in the LEAIM system [13,18,34,39,42,61,70,77].

## 7. Conclusions

Finally, the following results have been obtained.

(a) The following variations in the VLF signals propagation in the WGEI have been revealed:
  - a noticeable increase is in the spectrum at periods of 5–10 min; note that it covers the range of periods 6–7 min, which may indicate an effect on the modulation of the VLF EMW, propagating in the WGEI, caused by AGW oscillations near the Brunt–Väisälä period, i.e., a period of fundamental atmospheric fluctuations.
  - quasi-wave oscillations of the received signal with periods of 20–25 and 60–70 min, which can also be associated with AGWs, i.e., disturbances in the ULF range;
  - oscillations at periods of 3–4 h (probably, long-period gravity waves);
  Such disturbances can be excited by solar terminator, maybe earthquakes or other sources; the details of the proper excitation mechanism(s) will be a subject of the next paper(s).
  A weekly trend, which may be caused due to the effect of anthropogenic activities on the atmosphere/ionosphere, is also revealed.
(b) The very effective method of spatio-temporal characteristics, in particular, of VLF perturbations in the WGEI appeared to be the combination of detrending and wavelet analysis with proper averaging. At four different receivers from the network of Japan stations, oscillations of the same periods, in particular, 20 and 60 min., have been revealed. This procedure was successful, in particular, for the STU station only with detrending and after application of wavelet analysis, while without detrending the corresponding oscillations have not been seen;
(c) The information entropy has been found to show maxima near sunrise and sunset, and the time of these peaks relative to the indicated moments changes with season. The effect of solar flares on information entropy has been previously established as the VLF amplitude increase, but this issue needs further study;
(d) The presence of ULF modulation of the VLF EMW spectrum propagating in WGEI is qualitatively explained. The appearance of the combination frequency of VLF EMW and ULF AGW is due to the following effects: (1) the drag of charged plasma particles by ULF AGWs jointly with the background of VLF electron density disturbances and (2) the motion of charged plasma particles in the VLF EMW field jointly with the background of ULF changes in the plasma concentration caused by AGW. The features of the modulated VLF spectra found in the processing of experimental data are compared with the characteristics of various ULF oscillations of the atmosphere. They may be evanescent or reactive Lamb waves and global Brunt–Väisälä oscillations. The periods of such oscillations correspond to the spectral components that appear in the VLF EMW spectra in WGEI due to ULF modulation. These periods are 6–7 min, 20-60 min, and 3 h; the shorter period of ULF modulation of order 4 min (Figure 4n) may characterize the acoustic branch of AGW in the Earth–Thermosphere waveguide.

## Figures and Tables

**Table 1:** List of receiving stations in Japan.

Name of Receiving Station	Latitude φ [°]	Longitude λ [°]
AKT	40.10	140.08
ANA	33.90	134.67
IMZ	36.79	137.07
KMK	35.31	139.55
KTU	35.15	140.31
NSB	43.54	144.98
STU	42.80	140.23
TYH	34.74	137.37

## Data Availability

Not applicable.

## Appendix A

In this Appendix, we include two parts of the theoretical basis of the comparison between the revealed parts of ULF spectra, characterizing the modulation of VLF spectra in WGEI modulated by ionospheric response to AGW excitations: (a) basis of the influence of AGW on the ionosphere, mixing VLF EMW and ULF AGW frequencies (VLF EMWs modulation by AGWs); (b) basis of the most effective, namely resonantly excited AGW in the atmosphere. Corresponding spectra are used in the evaluations presented in the end of the Section 3 to compare our theoretical estimations and experimental data on the possible modulation by the ULF AGWs of VLF waves in WGEI.

(a) **Influence of AGW on the ionosphere and mixing VLF EMW and ULF AGW frequencies: VLF EMW modulation by AGWs**

Note that the aim of this Appendix is not to give the comprehensive theory of the ionospheric plasma response to AGW, but rather to show semi-qualitatively but quite clearly the fact of the existence of the oscillations on the combination of VLF and AGW frequencies. Therefore, the relations presented below are rather a qualitative illustration of this fact than the very detailed description of the process of the generation of the combined frequencies. The equations for the main two types of the charged particles (electrons and “averaged” ions) have the form [61,62].

(A1) $$N_{l}m_{l}\left[\frac{\partial {\vec{V}}_{l}}{\partial t}+\left({\vec{V}}_{l}\vec{\nabla }\right){\vec{V}}_{l}\right]-N_{l}\frac{q_{l}}{c}\left[{\vec{V}}_{l}\times {\vec{H}}_{0}\right]+\nu_{l}m_{l}N_{l}{\vec{V}}_{l}=N_{l}q_{l}m_{l}{\vec{E}}_{leff}\;$$

(A2) $${\vec{E}}_{leff}\equiv \vec{E}+\frac{\nu_{l}}{q_{l}}{\vec{v}}_{AGW}\;$$

(A3) $$\frac{\partial N_{l}}{\partial t}+\vec{\nabla }\left(N_{l}\vec{V_{l}}\right)=0\;$$

In the system (A1)–(A3), index l=i;e (indices “*i*” and “*e*” correspond to “averaged” ionospheric ions and electrons, respectively); $q_{i;e}$; $m_{l},N_{l},V_{l}$ and $\nu_{j}$ are the charge, mass, concentration, velocity, and effective collision frequency characterizing the charge particles characterizing by the index “*l*”, respectively; ${\vec{H}}_{0},\vec{E},{\vec{E}}_{jeff}$ and ${\vec{V}}_{AGW}$ are geomagnetic field, electric field in the ionosphere, effective electric field which influences on the charged particles characterizing by index “j”, accounting for the effect of dragging of charged particles by AGWs in the ionosphere and the velocity of neutral particles in the atmosphere–ionosphere system under the propagation of AGWs, respectively. Just to reveal qualitatively the main contributor of the VLF EMWs modulation by AGWs, we take apart the effects of wind and constant electric field, and note that the procedure of the linearization of the Equations (A1)–(A3) includes the presentation of the electron concentration in the form

(A4) $$N_{e}=N_{e0}+N_{e}^{\prime };N_{e}^{\prime }=N_{VLF}^{\prime }+N_{AGW}^{\prime }\;$$

In Equation (A4), $N_{e},N_{e0}$ and $N_{e}^{\prime }$ are the total electron concentration, steady-state (stationary) part of electron concentration, wave perturbation of electron concentration, the part of the electron concentration perturbation caused by VLF EMW in WGEI (on the frequency $\omega_{VLF}$ of VLF EMW) and the part of the electron concentration perturbation caused, due to dragging of charged particles, by ULF AGW (on the frequency $\omega_{AGW}$) in the ionospheric plasma of WGEI, respectively; the detailization of the form of expression for $N_{e}^{\prime }$ is not important for the qualitative description now and will be conducted in the next papers dedicated to the quantitative consideration of the VLF modulation by AGW in the WGEI; for VLF spectra even with the ULF modulation of EMWs in the WGEI, we could safely use the approximation of plasma quasineutrality, $N_{i}\approx N_{e}$ [62]. Note also that the system of the equations for the motion of charges particles (A1)–(A3) should be, generally speaking, supplemented by the system of equations for the neutral particles in the ionospheric plasma. At this stage, we suppose that the solution of the hydrodynamic equations for neutral atmosphere–ionosphere component [56,61,62,63] is presented in (A1) and (A2) by the AGW velocity ${\vec{V}}_{AGW}$, as a part of effective electric field, responsible for the dragging of the charged particles. Later in the present Appendix, we will stay on the spectrum characteristics of the resonant modes of AGW, the most interesting in the context of the interpretation of the VLF spectrum data presented in this paper. Accounting for the relations (A1)–(A4) and the equation of motion for neutral component (these equations are not presented here explicitly and can be found in [62,63], with inclusion of the effect of dragging of the neutral component by the plasma moving under the influence of VLF wave field, yields the presentation of the velocity and concentration for the plasma components in the form

$$N_{e}=N_{e0}+N_{AGW}^{\prime }+N_{VLF}^{\prime }\equiv N_{e0}+N_{AGW00}^{\prime }e^{j\omega_{AGW}t-jk_{AGW}}+$$

$$+N_{VLF00}^{\prime }e^{j\omega_{VLF}t-jk_{VFL}}$$

$$V_{AGW}=V_{AWG00}e^{j\omega_{AGW}t-jk_{AGW}}$$

(A5) $$\omega_{AGW}<<\omega_{VLF}\;$$

Hereafter, indices “AGW” and “VLF” in (A5) are used to indicate that the corresponding values are connected with ULF AGWs and VLF EMWs, respectively; indices “00” hereafter mean the complex amplitudes of the corresponding values. The amplitudes of the perturbations of the electron concentration and velocities of charged particles with the frequencies and wavenumbers of the AGW and VLF EMW in (A5), respectively, namely $N_{AGW00,VLF00}$, $V_{AGW00,VLF00}$ can be obtained using the standard linearization procedure [56,61,62,63] for the relations (A1)–(A3) and are not presented here. Accounting for (A1)–(A5), one can obtain for the ionospheric current $J^{VLF+AGW}$ on the mixed VLF EMW-ULF AGW frequency (to which upper index “VLF+AGW” corresponds), by the order of value, the following expressions

$$J^{\left(VLF+AGW\right)}={\left[\sum_{l=i,e}q_{l}N_{e}V_{l}\right]}^{\left(VLF+AGW\right)}∼$$

$$∼\{e\left({\hat{\mu }}_{iVLF}-{\hat{\mu }}_{eVLF}\right)N_{AGW00}^{\prime }{\vec{E}}_{VLF00}+$$

(A6) $$+\left(\nu_{i}{\hat{\mu }}_{iAGW}+\nu_{e}{\hat{\mu }}_{eAGW}\right)\cdot {\vec{V}}_{AGW00}\cdot N_{VLF00}^{\prime }\}e^{j[\left(\omega_{VLF}+\omega_{AGW}t-\left(k_{VLF+k_{AGW}y}\right)\right)]}\;$$

(A7) $$E_{VLF}=E_{VLF00}e^{j(\omega_{VLF}t-k_{VLF}y)};E_{VLF00}=A_{l}f_{E}\;$$

$$\hat{\epsilon }=\hat{I}+\frac{4\pi \hat{\sigma }}{i\sigma };\hat{\sigma }={\hat{\sigma }}_{i}+{\hat{\sigma }}_{e};$$

(A8) $${\vec{V}}_{i,e}={\hat{\mu }}_{i,e}{\vec{E}}_{eff};{\hat{\mu }}_{l}=\frac{1}{q_{l}N_{0e}}{\hat{\sigma }}_{l}\;$$

In (A6) and (A7) $E_{VLF},E_{VLF00},f_{E},A_{i}$ are the electric field of VLF EMW on the frequency $\omega_{VLF}$ without any perturbations (in particular, one caused by AGW), corresponding complex vector amplitude, corresponding polarization function with the proper altitude dependence and corresponding scalar complex amplitude, respectively. In (A8), $\hat{\sigma },\hat{\epsilon }$ and $\hat{\mu }$ are the tensors of ionospheric conductivity, dielectric permittivity, and mobility, respectively. Note that AGW can be considered as a factor that caused the slow (comparatively to the VLF characteristic time scales) variations of the ionospheric plasma in the WGEI. Similarly to (A6), it is possible to write the corresponding current varying in time with the difference of VLF EMWs and ULF AGWs frequencies (not shown here). The first and second terms in the last expression included in the right-hand part of (A6) correspond to the components of the combinational VLF-AGW frequency caused by (a) motion of charge particles in the VLF electric field on the background of the slow change of the ionospheric plasma entrained by the AGW and (b) motion of the charged plasma particles due to drag by AGW on the background of the variations of ionospheric concentration caused by VLF EMW, propagating in WGEI, respectively. For the system of coordinate connected with geomagnetic field (to which the index “(∼)” corresponds), the values of the mobility tensor components are of the form [61,62,78]

$${\hat{\mu }}_{i,e}^{\left(∼\right)}=\left(\begin{array}{ccc} \mu_{1\nu ,e} & \mu_{h\nu ,e} & 0\\ \mu_{h\nu ,e} & \mu_{1\nu ,e} & 0\\ 0 & 0 & \mu_{3\nu ,e} \end{array}\right)$$

$$\mu_{1\nu ,e}=\pm \frac{je(\omega -j\nu_{i,e})}{m_{i,e}\left[{\left(\omega -j\nu_{i,e}\right)}^{2}-\omega_{Hi,e}^{2}\right]\cdot \omega }=$$

$$\mu_{h\nu ,e}=-\frac{e\omega_{Hi,e}}{m_{i,e}\left[{\left(\omega -j\nu_{i,e}\right)}^{2}-\omega_{Hi,e}^{2}\right]}$$

(A9) $$\mu_{3i,e}=\mp \frac{je}{m_{i,e}\left(\omega -j\nu_{i,e}\right)};\omega_{Hi,e}=\frac{eH_{0}}{m_{i,e}c_{0}}\;$$

In the last one from the set of relations (A9), $c_{0}$ is the speed of light.

The current, $J^{\left(VLF+AGW\right)}$ in accordance with the theory of the waveguide excitation [79,80], generates the corresponding electromagnetic field on the combinational frequency of VLF and AGW in the WGEI, which, finally, is detected with a VLF receiver producing the data which are processed in the present paper. Using (A5)–(A8) and applying method NEELS (Nonlinear Evolution Equations for wave processes in Layered Structures) [20,57,70,81], one can find the electromagnetic field, including its electric component $E^{\left(AGW+VLF\right)}$ and and corresponding slowly varying amplitude $A^{\left(AGW+VLF\right)}$ on the combinational frequency $\left(\omega_{VLF}+\omega_{AGW}\right)$ (still lying in the VLF frequency range, as it follows from (A5)):

(A10) $$E^{\left(AGW+VLF\right)}∼A_{l}^{\left(AGW+VLF\right)}f_{E}e^{j[\omega_{VLF}+\omega_{AGW}t-\left(k_{VLFy}+k_{AGWy}\right)y]}\;$$

(the relation similar to (A10) can also be written for the corresponding magnetic field components). One of the goals of the present paper is to reveal the mixed AGW-VLF spectrum components in the VLF EMWs in WGEI. Any numerical evaluations of the corresponding amplitudes will be a subject of the next works. Here, we explain just qualitatively the physical basis of the occurrence of the mixed VLF EMW-ULF AGW frequencies (see relation (A10)) in the spectra of VLF EMW in WGEI. Therefore, the detailed derivation of the expressions for the $E^{\left(AGW+VLF\right)}$ and $A^{\left(AGW+VLF\right)}$ will be a subject of the next paper. We only note that, using (A6) and (A7), it is possible to obtain

(A11) $$\left|A_{l}^{\left(AGW+VLF\right)}\right|∼\left|A_{l}V_{AGW00}\right|\;$$

Note the following: The freedom of the interpretation of the relationship between the relative contributions from volume and surface currents (the last one is connected to the effective variations in the altitude of WGEI upper boundary caused by AGWs) into the generation of the mixed AGW-VLF frequencies in WGEI is connected with the very nature of this opened waveguiding structure. The consequence of this is the corresponding freedom of the choice of the altitude of the effective upper boundary of WGEI. If we choose this upper boundary to be high enough, the whole effect of the mixed frequency field generation can be reduced to the volume one, and we, in fact, have supposed that it is the case for the considered structure. Note also, concerning a possibility of the proper choice of an altitude of effective upper surface of the opened WGEI, the following: Our modeling based on the methods [61] shows that the density of energy flow in the direction of VLF EMW propagation decreases by (1.5–2) orders of value with increasing an altitude on the order of 10 km above z = 70 km. Therefore, the proper choice of an upper altitude of effective WGEI provides the possibility of using only the volume current for the qualitative consideration, in particular, of the effect VLF-AGW mixing. Alternatively, the VLF-AGW mixing can be presented as a result of the generation of the electromagnetic field at mixing VLF-AGW frequency caused by corresponding volume and surface currents [82]. Such a surface current can be presented in terms of oscillations with corresponding frequency of the effective upper waveguide boundary caused by AGWs [23,82]. Note again that the application of any of these approaches yields an occurrence of an electromagnetic field $E^{AGW+VLF}$ at the combined VLF-AGW frequency (see relations (A1)–(A10)).

It is supposed that VLF EMWs in WGEI, with given amplitude $A_{l}$, are incident in the region where plasma perturbations occur due to AGWs. Hence, to understand why the definite parts are revealed in the VLF spectra, as the corresponding ULF modulation, we will consider in the next parts of this Appendix which eigenmodes of the AGWs are revealed among all the all spectra of AGW excitations in the atmosphere and are the best candidates capable of providing the observed peculiarities of the spectra of ULF modulation of VLF waves.

(b) **The main evanescent (reactive) modes AGW in the atmosphere**

The system of equations for AGW in the isothermal lossless atmosphere [63] with sources in the forms of effective force ${\vec{F}}_{eff}$, effective mass production $R_{eff}$ and effective heating $Q_{eff}$ has the form [19,56,60].

(A12) $$\rho \frac{dV}{dt}+\vec{\nabla }p-\rho \vec{g}=\vec{F_{eff}};\;\vec{V}\equiv {\vec{V}}_{AGW}\;$$

(A13) $$\frac{d\rho }{dt}+\rho div\vec{V}=R_{eff}\;$$

(A14) $$\frac{\partial p}{\partial t}+\vec{V}\vec{\nabla }p-c_{0}^{2}\left(\frac{\partial \rho }{\partial t}+\vec{V}\vec{\nabla \rho }\right)=Q_{eff}\;$$

In Equations (A12)–(A14), ρ,p and $\vec{g}$ are atmospheric density and pressure and free-fall acceleration, respectively (index “AGW” is omitted hereafter for the sake of briefness). Without any details, we present the system (A14), after the linearization, in the form of matrix equation with the matrix ${\hat{D}}_{\rho p}$ (not included here explicitly) in its left-hand part, namely

$${\hat{D}}_{\rho ,p}\cdot \left(\begin{array}{c} V_{x}\\ V_{y}\\ \bar{\rho }\\ \bar{p} \end{array}\right)=\left(\begin{array}{c} F_{effx}\\ F_{effz}\\ R_{eff}\\ Q_{eff} \end{array}\right)$$

$$p_{0}=\rho_{0}gH$$

$$\rho_{0}=\rho_{00}e^{-z/H}$$

$$\bar{\rho }\equiv \rho^{\prime }/\rho_{0}\;\bar{p}\equiv p^{\prime }/p_{0}$$

(A15) $$\rho =\rho_{0}+\rho^{\prime };\;p=p_{0}+p^{\prime }\;$$

In (A15), $\rho_{00}$ is the atmosphere density at the ground level; $\rho_{0}$ and $p_{0}$ are the stationary values of the density and pressure in the stratified atmosphere, respectively. The eigenmode of the AGWs is determined by the dispersion equation

(A16) $$det\left({\hat{D}}_{\rho p}\right)=0\;$$

Of course, the effectiveness of AGW excitation depends not only on the properties of the matrix ${\hat{D}}_{\rho p}$, but also on the properties of the external sources included in the right-hand part of systems (A12)–(A15). The comprehensive approach to the AGW excitation in the atmosphere and corresponding ionospheric response (see item (a) in the present Appendix) will be developed and realized in the next papers. Now, we will restrict ourselves with the searching the global and resonant atmosphere modes among these determined by the solution of Dispersion Equation (A16), as it is noted in the text of our paper (see Section 3 in particular). This is important due to the global character of the sources (such as global terminator and X-ray flares), with the influence of these on the ionosphere being studied in the present paper; note that the global Lamb modes of the atmosphere are also excited effectively by the seismogenic sources, which are considered in the present paper as well, in accordance with [20,21]. To consider the roots of interest of the relation (A16), note that, in the absence of any sources in the system (A14), the roots of Equation (A16) coincide with the roots of the equation

$$A_{1}B_{2}-B_{1}A_{2}=0\;\mathrm{or}\;D_{t}^{4}+D_{t}^{2}\left[-\gamma gH\left(D_{y}^{2}+d_{z}^{2}\right)+\frac{\gamma g}{4H}\right]=\gamma^{2}\left(\gamma -1\right)D_{y}^{2}=0$$

$$A_{1}=D_{t}^{2}-\Gamma gHd_{y}^{2};B_{1}=\Gamma D_{y}\left(-\Gamma HD_{z}+1\right);A_{2}=\left[\left(\Gamma -1\right)g-\Gamma gHD_{z}\right]D_{y}$$

$$B_{2}=\left[d_{t}^{2}-\Gamma g\left(D_{z}H-1\right)\right]D_{y}$$

$$V_{y,z}∼exp\left[j\left(\omega t-k_{y}y-k_{z}\right)+1/2H\right];D_{t}=j\omega ;D_{y}\equiv -jk_{y}$$

(A17) $$D_{z}\equiv -jK_{z}\equiv -jk_{z}+1/2H\equiv d_{z}+1/2H\;$$

The coefficients $A_{1,2},B_{1,2}$ included in the dispersion equation in the form (A17) determine the polarization relations for AGWs:

(A18) $$A_{1}V_{y}+B_{1}V_{z}=0;A_{2}V_{y}+B_{2}V_{z}=0\;$$

From the dispersion equation presented in (A17), we present the characteristics of the global evanescent or reactive Lamb waves (see Equation (9), Section 3) and Brunt–Väisälä oscillations (Equation (10), Section 3) [20,55,56,57,58,59]. For the resonant Lamb waves, using (A17) and (A18), one can obtain

(A19) $$A_{1}=0,A_{2}=0;K_{z}=k_{z}+j/2H=j\left(\gamma -1\right)/\left(\gamma H\right)\mathrm{and}\left|v_{x}/V_{z}\right|\to \infty \;$$

For the Brunt–Väisälä oscillations, we obtain

(A20) $$A_{1}=0;B_{1}=0;K_{z}=k_{z}+j/2H=j/\left(\gamma H\right);\left|V_{x}/V_{z}\right|=\left|B_{2}/A_{2}\right|\;$$

Note also a principal possibility of existence of very interesting evanescent or reactive double resonant Brunt–Väisälä–Lamb modes [59] with

(A21) $$\omega =\omega_{BV}=c_{0}k_{y};\left|V_{x}\right|\to \inf;\left|V_{z}\right|\to \infty \;$$
